# Supplementary material for: The Hexane Extract of Citrus sphaerocarpa Ameliorates Visceral Adiposity by Regulating the PI3K/AKT/FoxO1 and AMPK/ACC Signaling Pathways in High-Fat-Diet-Induced Obese Mice
Source: Molecules. 2023 Dec 9;28(24):8026. doi: 10.3390/molecules28248026 (PMC10745821; doi:10.3390/molecules28248026)
Supplement: Supplementary file 1 [file molecules-28-08026-s001.zip › molecules-2738564-supplementary.pdf]

Supplementary Materials

Supplementary Table S1. Primer sequences used in this study.

Gene symbol	Accession Number	Forward primer (5'-3')	Reverse primer (5'-3')	Product size
Pparg	NM_011146.3	GCCCACCAACTTCGGAATC	TGCGAGTGGTCTTCCATCAC	57
Fasn	NM_007988.3	CCCTGACCAAGGTGCTGTTA	GGATCTCAGGGTTGGGGTTG	81
Srebf1	NM_011480	TATGGAGGGCATGAAACCCGAAG	TTGACCTGGCTATCCTCAAAG	165
Cebpa	NM_001287514.1	CGCAAGAGCCGAGATAAAGC	CGGTCATTGTCACTGGTCAACT	80
Cebpb	NM_001287738.1	AAGCTGAGCGACGAGTACAAGA	GTCAGCTCCAGCACCTTGTG	116
Ppara	NM_011144	GCCTGTCTGTCGGGATGT	GGCTTCGTGGATTCTCTTG	158
Acox1	NM_015729	CTTGGATGGTAGTCCGGAGA	TGGCTTCGAGTGAGGAAGTT	108
Ppargc1a	NM_008904.2	CAGTCGCAACATGCTCAAG	TGGGGTCATTTGGTGACTCT	73
Cpt1a	NM_013495	GATGTGGACCTGCATTCCTT	TCCTTGTAATGTGCGAGCTG	116
Gapdh	NM_001289726.2	GTTGTCTCCTGCGACTTCA	GGTGGTCCAGGGTTTCTTA	166

Supplementary Table S2. List of antibodies used in this study.

Protein Target	Name of Antibody	Supplier	Catalog No.	Host	Dilutions
AMPK	AMPKα1/AMPKα2 (Ab-183/172) Antibody	Signal way Antibody (SAB)	#21191	Rabbit	1:200
pAMPK	Phospho-AMPKα (Thr172) (D4D6D) Rabbit mAb	Cell Signaling Technology (CST)	#50081	Rabbit	1:200
ACC	Acetyl-CoA Carboxylase (C83B10) Rabbit mAb	CST	#3676	Rabbit	1:200
pACC	Phospho-Acetyl-CoA Carboxylase (Ser79) Rabbit mAb	CST	#11818	Rabbit	1:400
AKT	Akt (pan) (C67E7) Rabbit mAb	CST	#4691	Rabbit	1:300
pAKT	Phospho-Akt (Ser473) (D9E) XP® Rabbit mAb	CST	#4060	Rabbit	1:300
FoxO1	FOXO1 Recombinant Rabbit Monoclonal Antibody (SU33-01)	Invitrogen	MA5-32114	Rabbit	1:200
pFoxO1	Phospho-FOXO1 (Ser319) Polyclonal Antibody	Invitrogen	PA5-118569	Rabbit	1:200

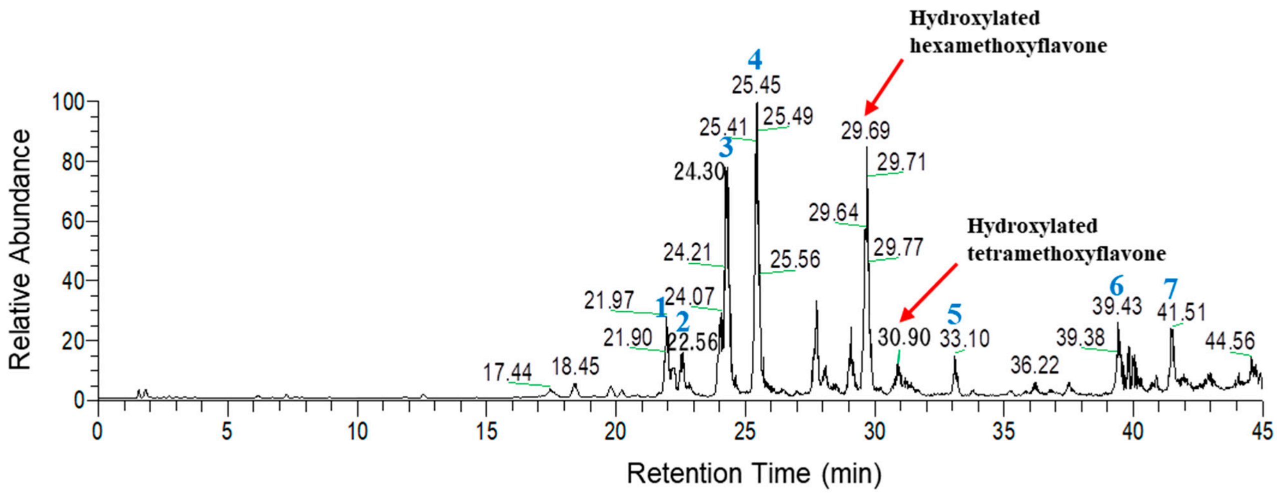

Figure S1. Total ion chromatogram of CSHE. Two peaks were detected at 29.69 and 30.90 min retention times, estimated as hydroxylated hexamethoxyflavone and hydroxylated tetramethoxyflavone by LC/MS analysis, respectively. The peak numbers show the following components: 1, naringenin, 2, nobiletin, 3, 3-methoxynobiletin [heptamethoxyflavone], 4, tangeretin, 5, hesperetin, 6, isosakuranetin, 7, auraptene.
